# Defining loneliness in older adults: protocol for a systematic review

**DOI:** 10.1186/s13643-018-0935-y

**Published:** 2019-01-17

**Authors:** Razieh Bandari, Hamid Reza Khankeh, Farahnaz Mohammadi Shahboulaghi, Abbas Ebadi, Abbas Ali Keshtkar, Ali Montazeri

**Affiliations:** 1 Nursing Department, University of Social Welfare and Rehabilitation Sciences, Tehran, Iran; 2 Health in Emergency and Disaster Research Center, University of Social Welfare and Rehabilitation Sciences, Tehran, Iran; 3 Department of Clinical Science and Education, Karolinska Institute, Solna, Sweden; 4 Clinical Psychology and Psychotherapy, Leipzig, Germany; 5 Iranian Research Center on Aging, Nursing Department, University of Social Welfare and Rehabilitation Sciences, Tehran, Iran; 6 Behavioral Sciences Research Center, Life Style Institue, Nursing Faculty, Baqiyatallah University of Medical Sciences, Teheran, Iran; 7 Department of Health Sciences Education Development, School of Public Health, Tehran University of Medical Sciences, Tehran, Iran; 8 Mental Health Research Group, Health Metrics Research Centre, Iranian Institute for Health Sciences Research, ACECR, Tehran, Iran; 9 Faculty of Humanity Sciences, University of Science & Culture, ACECR, Tehran, Iran

**Keywords:** Loneliness, Elderly, Older adults, Systematic review, Definition

## Abstract

**Background:**

Socialization is an important part of the healthy aging process, but natural changes in the lifestyle and health of older people increased risk of loneliness. However, loneliness is not well defined and might differ in different cultures and settings. The main objective of this systematic review is to summarize literature on the topic and propose a definition that might help aging research and practice in the future.

**Methods:**

Eight databases including PubMed, Scopus, CINAHL, Web of Science, EMBASE, PsycINFO, Proquest, and Age Line bibliographic will be run individually to retrieve relevant literature on loneliness among elderly population using subject headings and appropriate MeSH terms. Inclusion and exclusion criteria will be developed and refined by the research team. Two reviewers will participate in each search stage including abstract/title and full text screening, data extraction, and appraisal. We will restrict our search to articles published in the English language biomedical journal between 2000 and 2017. The protocol adheres to the standards recommended by the PRISMA-P.

**Discussion:**

The results of this systematic review can present a more accurate definition of loneliness for researchers who aim at conducting new primary and secondary studies on this subject.

**Systematic review registration:**

CRD42017058729

**Electronic supplementary material:**

The online version of this article (10.1186/s13643-018-0935-y) contains supplementary material, which is available to authorized users.

## Background

Improved life conditions, higher longevity, and increased life expectancy have made aging a dominant phenomenon in communities leading to rise of elderly population. It is estimated that, in 40 years’ time, the population of people above 60 years of age will be doubled in the world [1]. Thus, the ratio of people above 60 years to the rest of the world population (which was 11% in 2006) will reach to 22% in 2050 [2–5]. It is also predicted that the proportion of old people in developing and developed countries will reach to 80% and 40%, respectively [4, 6–9]. This rising has become a serious economic, social, and health challenge for health care providers, family members, and societies in the twenty-first century [10, 11].

Apparently, the nature of old age provides conditions for feeling lonely. In other words, it puts individuals in conditions under which people feel lonelier [12, 13]. Loneliness is an internal, unpleasant subjective experience that begins when an individual’s social network undergoes a qualitative or quantitative loss [14–17]. Loneliness is different from being alone or living alone. In fact, a person may suffer from feeling of loneliness even in the presence of other people. On the contrary, an individual may live alone but does not feel lonely [18, 19]. Some studies have reported that the prevalence of loneliness in the elderly in various European countries range from 3 to 34%. It has also been demonstrated that loneliness is lower among North European countries such as Denmark, Germany, Sweden, and Britain compared to other countries in Eastern and Southern Europe [20]. It is estimated that as high as one-third of the elderly experience some degree of loneliness at the end of their life [21–23]. Even older adults face an elevated risk of loneliness as they age, especially those aged 80 and over [24]. As suggested, about 50% of those aged 80 and over report frequent feelings of loneliness [25]. However, this high prevalence indicates that loneliness is a serious threat for the elderly population and must be taken seriously.

It has been suggested that several factors might contribute to loneliness among elderly people. A review of the available literature illustrates that spouse loss, declining health, reduced social relations, and hospitalization are some of the factors that worsen loneliness among the elderly [26, 27]. From another perspective, loneliness among the elderly can be attributed to increasing functional disability and decreasing social contacts [28, 29]. Pinquart and Sorensen carried out a meta-analysis and found that loneliness was associated with age, gender (females feel lonelier), socioeconomic status (individuals with lower socioeconomic status feel lonelier), and place of living (people who live in nursing homes are lonelier) [30]. Yan and Yang conducted a cross-temporal meta-analysis focusing on the rising feeling of loneliness among Chinese elderly between 1995 and 2011. They reported that factors such as urban life, divorce rate, and unemployment rate influenced the degree of feeling lonely among Chinese elderly [22]. In fact, the findings suggest that keeping traditional ways of life and working toward minimizing the divorce rate would result in better social integration and less loneliness of older adults. Perhaps other pertinent goals should be to improve quality of life for older people.

Loneliness can cause physical and mental disorders experienced by the elderly [18, 31]. Various studies have indicated that loneliness will have serious health-related consequences including depressive symptoms, cognitive decline, intense feelings of emptiness, abandonment, frequent visit of doctors, and poorer quality of life [32–34]. Loneliness also increases the risk of committing suicide [35].

Having read the literature, one might argue what constitutes loneliness above all? Various definitions of loneliness have been proposed in different studies. According to one definition, loneliness is a complex mental excitement which is generally experienced in the form of an unpleasant feeling of stress due to lack of connection or commonality with others [14]. Based on another definition, loneliness is a complicated feeling with psychological and social dimensions and is regarded as an important indicator of well-being in humans [36]. Perlman and Peplau believe that loneliness is a mental, unpleasant, and distressing phenomenon that is the result of inconsistency between individuals’ expected level of social relations and the real level of connections that is exercised [16]. It is thus argued that loneliness is the outcome of inconsistency between expected strength of social networks and the quality of these relations that is experienced in reality [17].

The concept of loneliness was initially studied in disciplines such as psychology and social work [37]. In these studies, loneliness was conceptualized existentially, pathologically, and sociologically as a depressive symptom [38, 39]. However, later on, health scholars and those who involved in geriatrics touched the topic and proposed different descriptions for loneliness among elderly populations. For instance, in a study conducted by Karki (2009), the concept of loneliness was investigated among old women. Accordingly, loneliness was divided into five categories including marital status (being a widow or not), health condition, immigration, lifelong single hood, and social isolation [40]. However, currently, researchers are trying to further clarify the definition of this concept (i.e., loneliness) since understanding this notion from old people’s perspective can enhance health care and rehabilitation services, preventing it, and providing necessary interventions [37]. Without a correct understanding of concepts, various phenomena cannot be appropriately explained and professionals will not be able to share a common language [41]. Analyzing the concept of loneliness from the elderly’s viewpoint can help us to come up with essential components and guidelines that may influence clinical research and practice. However, studies on the concept of loneliness among elderly are very limited. Most studies only focus on the strength of this feeling and its correlates. Therefore, the current study aimed to come up with a comprehensive description of loneliness among elderly by investigating available literature. Of particular importance to this study is addressing the following three research questions:

1. What definitions have been proposed for loneliness among the elderly in the geriatric literature?
2. Which definition is more comprehensive and executable?
3. Are there any variations in defining loneliness between developing and developed settings?

## Objectives

### Primary objective

The main aim of this study is to summarize the definition of loneliness.

### Secondary objectives

1. Proposing a comprehensive definition for loneliness
2. Assessing the heterogeneity of definitions among different primary research according to developed versus developing countries, and loneliness prevalence (high prevalence versus low prevalence)

## Methods

### Eligibility criteria

In this systematic review, the researchers intend to include and investigate the findings of case series, cross-sectional, case-control, cohort, interventional (field trials, community interventional trials, randomized or non-randomized clinical trials, true experimental, or quasi-experimental studies), qualitative studies, and review articles giving that the studies report at least one definition of sense of loneliness. Studies that have concentrated on animals will not be included.

### Participants

This study includes research projects in which all or some groups of participants are older adults aged 60 years and over. However for developing countries, 50 years will be considered since the World Health Organization defines age 50 and over as elderly [42].

### Setting and time frame

This systematic review encompasses both clinical (conducted in hospitals or nursing centers) and social studies.

### Report characteristics

We just include papers that have an English abstract. There is no limitation in terms of date of acceptance or publication. With respect to publication status, we only consider papers that have been published or are in press.

### Exclusion criteria

The following studies will be excluded:

- Studies that published in languages other than English
- Abstract, conference abstracts, book, book chapters, protocols, editorials letter will be excluded.

### Information sources

Information resources will comprise the following categories: electronic databases, databases of clinical trials, different types of gray literature, researchers, and authors. Electronic search will be carried out in the following databases: PubMed, Scopus, CINAHL, Web of Science, EMBASE, PsycINFO, and AgeLine bibliographic. Medical Subject Headings (MeSH) and popular expressions in the related literature will be used as keywords. Search strategy will be primarily developed and completed in PubMed. Then, the same strategy will be pursued in other databases. Other resources will also be investigated to identify the gray literature. Moreover, thorough search will be conducted in Proquest to find relevant theses and dissertations. Seminar abstracts will be studied via Scopus, Web of Science, and other related websites. The reference lists of published papers and systematic reviews and the table of contents of key journals in this area will also be investigated. The review will include studies that were conducted/published between 2000 and 2017, hence being able to track general trends in the definition of loneliness in various periods.

### Search strategy

Our initial search syntax for PubMed will be (loneliness [tiab] OR homesickness [tiab]) AND (old [tiab] OR old*[tiab] OR eld* OR geriatric* OR aging OR age* OR “later life” OR senior OR nonagenarian OR octogenarian OR centenarian) AND (2000/01/01:2017/12/31[dp] (Additional file 1).

### Selection process

The two authors will independently screen the collected papers in the first step. Each of them will first review the title and abstracts of the papers, followed by classifying the selected papers into three groups: related, unrelated, and undecided papers. The papers that are classified as “unrelated” will be removed from further analysis. Then, the two researchers will go over the remaining papers independently trying to come up with a list of papers that must be included in the review. The two lists will be compared and areas of dispute will be resolved through discussion. In cases of disagreement, the entire team makes the final decision about the inclusion (or exclusion) of a paper. The Cochrane Public Health Group’s manual will be used to extract quantitative studies [43]. The extraction of qualitative studies will be based on a form that was developed by the Joanna Briggs Institute [44].

### Data management

The two researchers will independently extract the related data from the collected papers and will be recorded in data sheets. A third party will review the two data sheets. Possible disagreements between the two researchers will be discussed with the whole team. If no solution is obtained, the researchers will contact the authors of the paper to make the final decision (Additional file 2).

### Data items

The following information will be collected from each paper: author(s), year of publication, journal title, format (summary or journal paper), design and setting, country, study objective(s), theory and/or hypothesis, definitions of loneliness, instruments for data collection, samples’ demographic information (age, gender, etc.), type of participants, sample size, geographical domain, and time of data collection.

### Risk of bias in individual studies

Risk of bias analysis will involve exploration of limitations and appropriateness of study methods in addressing their research questions and objectives, and how they inform outcomes. Particularly, studies will be critically assessed for their design, data collection and analysis methods, selection bias, integrity, confounders, attrition, and reporting. Thereafter, we will categories and summaries the findings uncertain, high, or low [45]. The Cochrane Collaboration tool for risk bias will be used in the assessment of controlled trials [46]. Risk of bias in non-randomized studies of interventions (ROBINIS-I) tool will be used to assess all other quantitative studies such as non-controlled trials and quasi-experiments [47]. The dependability of qualitative studies will be appraised with a form that was developed by the Joanna Briggs Institute namely the Qualitative Assessment and Review Instrument [44].

### Data synthesis

In the final report, we will present a domain of definitions in the form of a list with various subgroups. The papers will be divided into these subgroups on the basis of their evidence, type of participants (inpatients, outpatients, general population, etc.), and the context of the study (e.g., hospital, clinic, society, etc.). Finally, we will record various definitions of loneliness in the elderly in tables of results. These definitions will be recorded on the basis of their importance and the degree of satisfaction as determined by their psychological quality, the rigidity of the findings, and the amount of available evidence. Various definitions will then be compared and contrasted. The collected data will also be used to demarcate various chronological trends in defining loneliness. Subsequently, the data will be combined and categorized based on the procedure mentioned above. The final report will be prepared based on the Preferred Reporting Items for Systematic Reviews and Meta-Analyses (PRISMA).

## Discussion

Over the past two decades, numerous observational and interventional studies have investigated the concept of loneliness. Nevertheless, this important health problem still suffers from the lack of a unique and accurate definition. Thus, all the reviews conducted in this area are inconsistent because they have focused on various research samples, research designs, and contexts. Because of inconsistent definitions of loneliness, these reviews are biased studies to one degree or another. The results of this systematic review can present a more accurate definition of loneliness for researchers who aim at conducting new primary and secondary studies on this subject. Furthermore, the results can have an important role in improving the internal consistency of future evidence.

### Strengths and limitations

This systematic review develops a consensus on the definition of loneliness, a controversial term in medical articles. Two investigators with extensive experience in systematic review independently will carry out primary screening of the articles, data extraction, and quality assessment in order to minimize the probability of personal biases. However, the review will not include databases in languages other than English (French, German, Chinese, etc.). This limitation may cause language bias.

## Additional files

Additional file 1: The final syntax. (DOC 27 kb)

Additional file 2: The schematic presentation of the selection process of articles for final systematic review. (DOC 40 kb)

## Abbreviations

CINAHL: Cumulative Index to Nursing and Allied Health Literature
EMBASE: Excerpta Medica database
MeSH: Medical subject headings
PQDT: The ProQuest Dissertations & Theses Database
PRISMA: Preferred Reporting Items for Systematic Reviews and Meta-Analyses
PROSPERO: International Prospective Register of Systematic Reviews
PsycInfo: Psychological Information Database
ROBINIS-I: Risk of bias in non-randomized studies of interventions
