# Supplementary material for: Defining loneliness in older adults: protocol for a systematic review
Source: Syst Rev. 2019 Jan 17;8:26. doi: 10.1186/s13643-018-0935-y (PMC6335854; doi:10.1186/s13643-018-0935-y)
Supplement: Supplementary file 1 — The final syntax. (DOC 27 kb) [file 13643_2018_935_MOESM1_ESM.doc]

**Final syntax in SCOPUS:**

(TITLE-ABS(loneliness*) OR TITLE-ABS(homesickness)) AND (TITLE-ABS(old) OR ALL(old*) OR ALL(eld*) OR ALL(geriatric*) OR ALL(aging) OR ALL(age*) OR ALL("later life") OR ALL(senior) OR ALL(nonagenarian) OR ALL(octogenarian) OR ALL(centenarian)) AND (PUBYEAR < 2018 AND PUBYEAR > 1999)

**Final syntax in WOS**

(TS=(loneliness*) OR TS=(homesickness)) AND (TS=(old) OR TS=(old*) OR TS=(eld*) OR TS=(geriatric*) OR TS=(aging) OR TS=(age*) OR TS=(“later life”) OR TS=(senior) OR TS=(nonagenarian) OR TS=(octogenarian) OR TS=(centenarian))

**EMBASE syntax:**

loneliness:ab,ti OR homesicknes:ab,ti AND (old*:ab,ti OR eld* OR geriatric* OR 'aging'/exp OR aging OR age* OR 'later life' OR senior OR 'nonagenarian'/exp OR nonagenarian OR 'octogenarian'/exp OR octogenarian OR 'centenarian'/exp OR centenarian) AND [2000-2017]/py

**Psycinfo with OVID syntax:**

(loneliness or homesicknes).ab,ti. and (old*.ab,ti. or (eld* or geriatric* or aging or age* or "later life" or senior or nonagenarian or octogenarian or centenarian)).af.

**Agelinesyntax:**

(loneliness* OR homesickness) AND (old OR old* OR eld* OR geriatric* OR aging OR age* OR "later life" OR senior OR nonagenarian OR octogenarian OR centenarian) AND PY(2000-2017)

**CINAHL syntax:**

(loneliness* OR homesickness) AND (old OR old* OR eld* OR geriatric* OR aging OR age* OR "later life" OR senior OR nonagenarian OR octogenarian OR centenarian) AND PY(2000-2017)
