# Supplementary material for: Defining loneliness in older adults: protocol for a systematic review
Source: Syst Rev. 2019 Jan 17;8:26. doi: 10.1186/s13643-018-0935-y (PMC6335854; doi:10.1186/s13643-018-0935-y)
Supplement: Supplementary file 2 — The schematic presentation of the selection process of articles for final systematic review. (DOC 40 kb) [file 13643_2018_935_MOESM2_ESM.doc]

Additional file 2

| Potentially relevant studies and publications obtain through database searching activities and email (n)  Additional studies collected using other relevant sources (email request, thesis/dissertation and other data sharing mechanisms)  Number of studies after removal of duplications  Papers excluded on the basis of title (n)  Number of studies included based on abstract (n)  Papers eligible for the whole body review for eligibility to be included to the final review (n)  Papers excluded based on reviewing the abstract parts (n(  Exclude from the review after assessing the full text of the paper. The papers did not clearly reported prevalence and associated factors (n)  Relevant information to be systematically review and extracted (n)  Number of studies used in the qualitative synthesis (systematic review)  Number of studies used for quantitative meta-analysis  Figure S1: diagramatic presentation of the selection process of artilces for final systeamtic review |
| --- |
|  |
